
JOURNAL INFORMATION
==============================
NLM Title Abbreviation: Microbiologyopen
Journal ID: Microbiologyopen
NLM Journal ID: 101588314
Journal ID: mbo3

MicrobiologyOpen

EISSN: 2045-8827
Publisher: Wiley

ARTICLE INFORMATION
==============================
PMCID: PMC4082701
DOI: 10.1002/mbo3.131
Article version: 1
Subjects: Issue Information

Issue Information

Print publication date: 2014 Jun
Electronic publication date: 2014 Jun 10
Volume: 3
Issue: 3
First page: i
Last page: iii
Copyright: © 2014 Published by John Wiley & Sons Ltd
Copyright year: 2014
License: This is an open access article under the terms of the Creative Commons Attribution License, which permits use, distribution and reproduction in any medium, provided the original work is properly cited.
License URL: https://creativecommons.org/licenses/by/3.0/

==============================
Volume 3, Number 3

### MicrobiologyOpen

Open Access

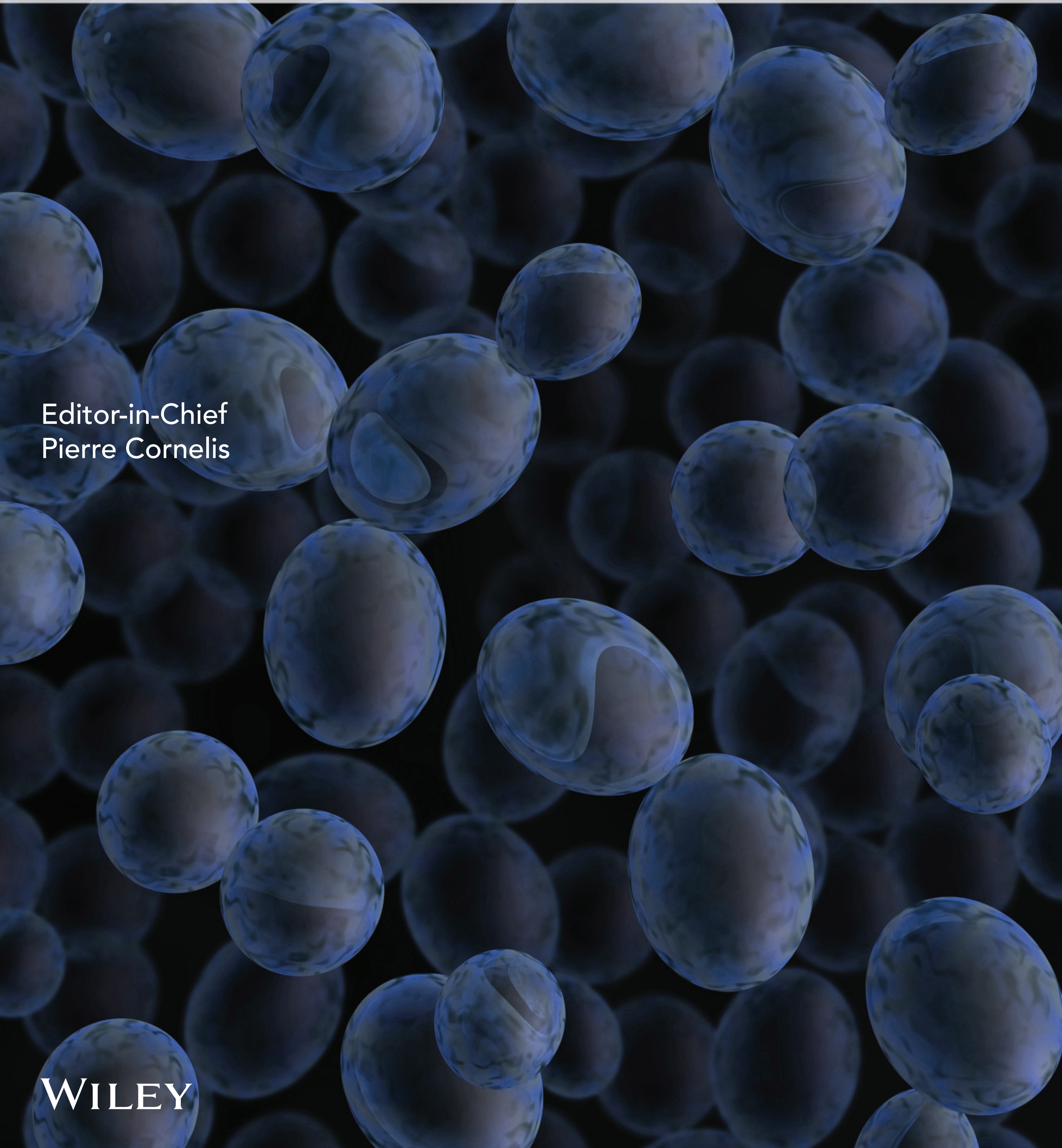

Editor-in-Chief
Pierre Cornelis

WILEY

MicrobiologyOpen

Open Access

### Aims and Scope

*MicrobiologyOpen* is a peer-reviewed journal delivering rapid decisions and fast publication of microbial science, a field which is undergoing a profound and exciting evolution in this post-genomic era. The journal gives priority to reports of quality research, pure or applied, that further our understanding of microbial interactions and microbial processes. *MicrobiologyOpen* gives prompt and equal consideration to papers reporting theoretical, experimental, applied and descriptive work in all aspects of microbiology.

The journal aims to serve there search community by providing a vehicle for authors wishing to publish interesting and high quality work in both fundamental and applied microbiology. *MicrobiologyOpen* will consider submissions across eukaryotic microorganisms, prokaryotes (bacteria and archaea) and viruses infecting or interacting with microorganisms, including genetic, biochemical, biophysical, bioinformatic and structural analyses.

The journal features original research articles, reviews and editorials. Original research papers must report well-conducted research with conclusions supported by the data presented in the paper.

*MicrobiologyOpen* publishes papers submitted directly to the journal and those referred from a select group of prestigious journals published by Wiley-Blackwell.

*MicrobiologyOpen* is a Wiley Open Access journal, one of a new series of peer-reviewed titles publishing quality research with speed and efficiency. For further information visit the Wiley Open Access website at http://www.wileyopenaccess.com.

### Open Access and Copyright

All articles published by *MicrobiologyOpen* are fully open access: immediately freely available to read, download and share. All articles accepted from 14 August 2012 are published under the terms of the Creative Commons Attribution License. All articles accepted before this date were published under a Creative Commons Attribution Non-Commercial License. The Creative Commons Attribution License permits use, distribution and reproduction in any medium, provided the original work is properly cited and allows the commercial use of published articles. Copyright on any research article in a journal published by *MicrobiologyOpen* is retained by the author(s). Authors grant Wiley a license to publish the article and identify itself as the original publisher. Authors also grant any third party the right to use the article freely as long as its integrity is maintained and its original authors, citation details and publisher are identified. Further information about open access license and copyright can be found at http://www.wileyopenaccess.com/details/content/12f25db4c87/Copyright–License.html.

### Purchasing Print Reprints

Print reprints of Wiley Open Access articles can be purchased from corporatesales@wiley.com.

### Disclaimer

The Publisher and Editors cannot be held responsible for errors or any consequences arising from the use of information contained in this journal; the views and opinions expressed do not necessarily reflect those of the Publisher and Editors, neither does the publication of advertisements constitute any endorsements by the Publisher and Editors of the products advertised. Wiley Open Access articles posted to repositories or websites are without warranty from Wiley of any kind, either express or implied, including, but not limited to, warranties of merchantability, fitness for a particular purpose, or non-infringement. To the fullest extent permitted by law Wiley disclaims all liability for any loss or damage arising out of, or in connection with, the use of or inability to use the content.

**Editor-in-Chief**

Pierre Cornelis
*Email: pierre.cornelis@microbiologyopen.com*
*Address correspondence to the Editorial Office: oa-microbiology@wiley.com*
*Tel.: +44(0) 1865 476302*

**Editorial Board**

Sonja-Verena Albers
*Max-Planck-Institute for Terrestrial Microbiology, Germany*

Simon C. Andrew
*University of Reading, UK*

Scott Beatson
*University of Queensland, Australia*

Wilbert Bitter
*University of Amsterdam, The Netherlands*

Jesús Blazque
*CSIC, Spain*

Eric Cascalès
*Université Aix-Marseille, France*

Jennifer Cavet
*University of Manchester, UK*

Daniel Charlier
*Vrije Universiteit Brussel, Belgium*

Victor J. Cid
*Universidad Complutense de Madrid, Spain*

Eric Dézièl
*INRS-Laval, Canada*

Steve Diggle
*University of Nottingham, UK*

Alan Dobson
*University College Cork, Ireland*

Friedrich Götz
*University of Tübingen, Germany*

Emmanuel Lesuisse
*Institut Jacques Monod, France*

Roland Lloubès
*Université Aix-Marseille, France*

Jose L. Martinez
*CSIC, Spain*

Skorn Mongkolsuk
*Mahidol University, Thailand*

Cheryl A. Nickerson
*Arizona State University, USA*

Mathieu Picardeau
*Institut Pasteur, France*

Wim J. Quax
*University of Groningen, The Netherlands*

Jacques Ravel
*University of Maryland, USA*

Edson Rocha
*East Carolina University, USA*

Fernando Rojo
*CSIC, Spain*

Lígia Saraiva
*Instituto de Tecnologia Química e Biológica, UNL, Portugal*

Isabelle Schalk
*ESBS, France*

Konstantin Severinov
*Rutgers University, USA*

Jonathan Shaw
*University of Sheffield, UK*

Stephen Spiro
*University of Texas at Dallas, USA*

Alain Stintzi
*University of Ottawa, Canada*

Mark S. Thomas
*University of Sheffield, UK*

Miguel A. Valvano
*University of Western Ontario, Canada*

Laurence Van Melderen
*Université Libre de Bruxelles, Belgium*

Cynthia Whitchurch
*University of Technology, Sydney, Australia*

Liping Zhao
*Shanghai Jiao Tong University, China*
